# Sex Steroids and Adult Neurogenesis in the Ventricular-Subventricular Zone

**DOI:** 10.3389/fendo.2018.00156

**Published:** 2018-04-09

**Authors:** Giovanna Ponti, Alice Farinetti, Marilena Marraudino, GianCarlo Panzica, Stefano Gotti

**Affiliations:** ^1^Department of Veterinary Sciences, University of Turin, Grugliasco,Turin, Italy; ^2^Neuroscience Institute Cavalieri Ottolenghi (NICO), Orbassano, Turin, Italy; ^3^Department of Neuroscience “Rita Levi-Montalcini”, University of Turin, Turin, Italy

**Keywords:** ventricular-subventricular zone, sexual dimorphism, estrogens, testosterone, neural stem cells, puberty, estrous cycle, pregnancy

## Abstract

The forebrain ventricular-subventricular zone (V-SVZ) continuously generates new neurons throughout life. Neural stem cells (type B1 cells) along the lateral ventricle become activated, self-renew, and give rise to proliferating precursors which progress along the neurogenic lineage from intermediate progenitors (type C cells) to neuroblasts (type A cells). Neuroblasts proliferate and migrate into the olfactory bulb and differentiate into different interneuronal types. Multiple factors regulate each step of this process. Newly generated olfactory bulb interneurons are an important relay station in the olfactory circuits, controlling social recognition, reproductive behavior, and parental care. Those behaviors are strongly sexually dimorphic and changes throughout life from puberty through aging and in the reproductive age during estrous cycle and gestation. Despite the key role of sex hormones in regulating those behaviors, their contribution in modulating adult neurogenesis in V-SVZ is underestimated. Here, we compare the literature highlighting the sexual dimorphism and the differences across the physiological phases of the animal for the different cell types and steps through the neurogenic lineage.

## Introduction

**Figure 1:**
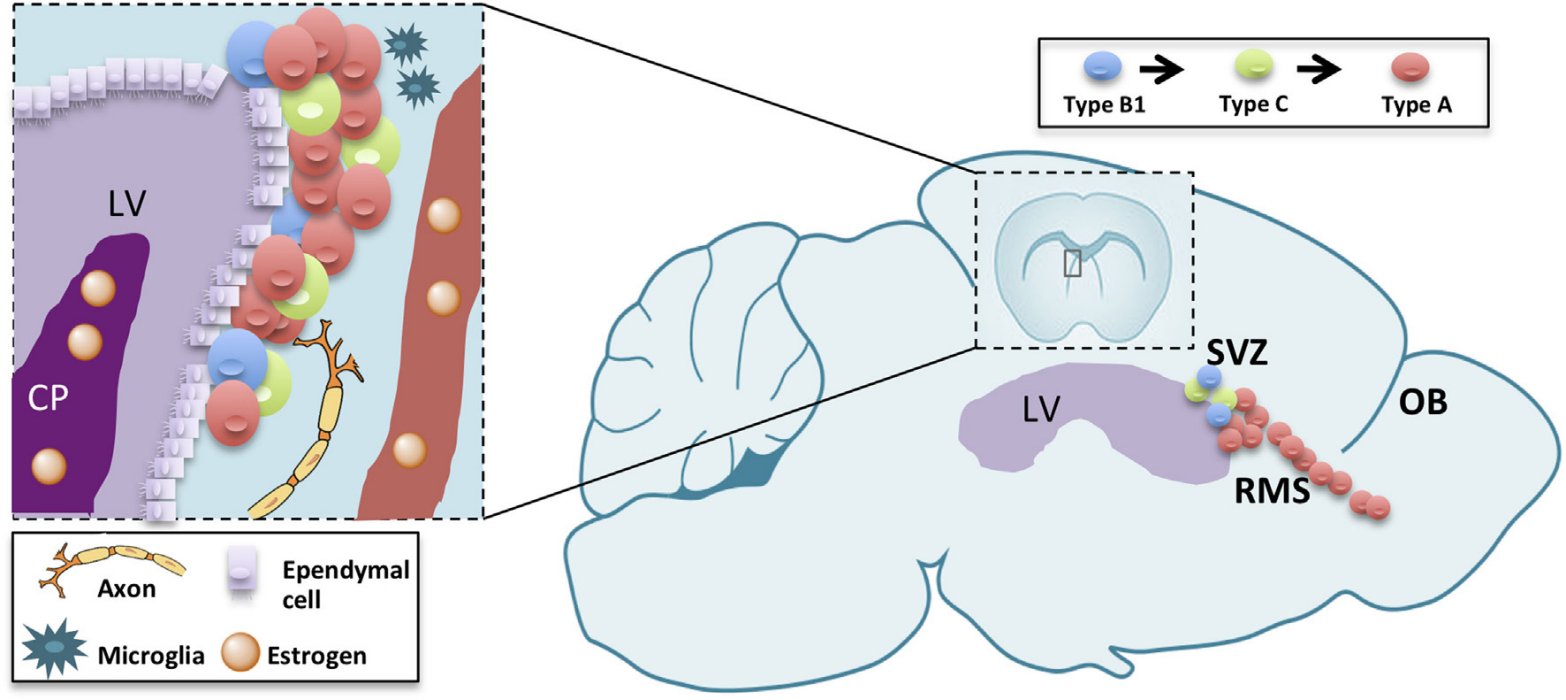
Schematic drawing summarizing adult neurogenesis in the V-SVZ/OB system. Adult neurogenesis is a multiple-step process, occurring in three different subregions: the ventricular-subventricular zone (V-SVZ), the rostral migratory stream (RMS), and the olfactory bulb (OB). Sex hormones reaching the lateral ventricle (LV) through the choroid plexus (CP) or blood vessels (BV) modulate each of those steps either directly on neurogenic lineage or indirectly through other component of the stem-cell niche or the parenchyma.

The subventricular zone-olfactory bulb (V-SVZ-OB) system has fascinated scientists for over than 25 years. In fact, this region harbors, in many mammals, a huge neurogenesis persisting until aging (1). In rodents, this process involves multiple steps, each one of them representing a model for different biological and pathological processes with unique features. In fact, this neurogenic process encompasses a germinal layer located in the ventricular-subventricular zone of the forebrain (V-SVZ), along the ventricle in which neural stem cells undergo self-renewal (2) and differentiation to intermediate progenitors (type C cells), then to immature neurons (type A cells) (3–5). Newly generated, type A, cells undergo tangential migration along the rostral migratory stream (RMS) up to the OB (6, 7). There, they migrate radially to the appropriate cell layer and differentiate into interneurons (8). Neurogenesis is thus a complex process consisting in proliferation, migration, apoptosis, and differentiation occurring in each of those levels with specific features (9, 10). The proper turnover enforced by proliferation, migration as well as apoptosis in the OB, is essential for optimizing olfaction [(11); Figure 1]. Therefore, the study of V-SVZ is capital for many purposes: understanding unregulated cell growth in tumor formations (12, 13), preventing or replacing cell loss in aging (1, 14, 15), decreasing neurodegenerative disease risks (16–18), and improving stroke treatments (18).

Despite a huge interest on the endogenous and exogenous factors affecting adult neurogenesis in V-SVZ-OB system (19), few studies have focused on the role of gonadal hormones. This flaw is surprising since steroids have a key role in hippocampal neurogenesis both during development and in adulthood (20–22). Furthermore, V-SVZ-OB system is involved in social and reproductive behaviors, which are strongly regulated by sexual steroids (23, 24) and are targets for xenoestrogens (25–27).Moreover, estrogen receptors (ERs) and enzymes involved in the biosynthesis of steroids such as aromatase, the enzyme converting testosterone (T) into estradiol, are expressed in the V-SVZ (28) and in the OB of adult (29, 30) and developing (31) rats and mice (32). However, while the importance of steroids in the regulation of adult neurogenesis in the hippocampus has been widely studied, its role in the V-SVZ-OB system is more debated. Here, we want to focus on the available data in order to encourage a discussion addressing the open questions in the field.

## Sexual Dimorphism in V-SVZ-OB System

Sexual dimorphism in the V-SVZ-OB system is an open question. Only few studies compared the two sexes and most of them are limited to a few ages. Indeed, the extent of neurogenesis in this region changes along life and it is likely to be affected by changes in the endocrine system.

Neurogenesis is more prominent in adult female mice compared with males. In 3-month-old C57/BL6J mice, females displayed higher proliferating rates in V-SVZ, RMS, and OB, and lower apoptotic cells in V-SVZ in both estrus and pregnancy (33) than males. Similarly, the number of neuronal progenitors (SOX2+) in the V-SVZ of females was higher than males in young adults but not in pups (34).

On the other hand, in other studies, the density of apoptotic cells in accessory (AOB) and main (MOB) OB was similar in the two sexes (35). Some differences affect transiently specific features of the V-SVZ-OB system. For instance, peripubertal males displayed higher rates of apoptosis (33), as well as of proliferation in the V-SVZ compared with females (35), but, in 2-month-old animals, the proliferation rate in the V-SVZ is similar in the two sexes and 1 month later there was a similar supply of newly generated cells in both the MOB and the AOB (35).

Multiple factors may explain the discrepancy among the data. From a technical point of view, the methods used to assess cell proliferation may highlight a different subset of the cycling population. In fact, while the total number of cycling cells identified with PCNA was measured by Diaz (33), the study of Nunez-Parra (35) highlighted only the cells in the S-phase, labeled by BrdU 2 h after the injection of the marker. Thus, it may reflect differences in the cell-cycle length between the two ages, or differences in the composition of the V-SVZ, e.g., a decrease in the number of type C cells, which have a longer S-phase length compared with type A cells (4, 5) or even differential sensitivities of BrdU antibodies (36), although the use of two different anti-BrdU antibodies by Nunez-Parra et al. is likely to have decreased this issue. Moreover, since different subregions in the V-SVZ give rise to different interneurons in the OB (37), it is possible that sexual dimorphism is limited to some of them. In addition to that, the extent of neurogenesis is dissimilar in different mouse strains (38) and it might be differently regulated. In fact, other reports indicate that the higher number of proliferating cells in the V-SVZ of females is limited to mature animals, i.e., 6–8 months old (39). Interestingly, this dimorphism is abolished (SJL/J) or reverted (BALB/C) in different strains (39). Accordingly, in two months old C57BL6 mice, the density of newly generated cells is higher in the AOB of males than females, while no sexual dimorphism has been reported for MOB (34). Similarly, no sexual dimorphism was observed in the number of newly generated cells in the AOB of young-adult CD1 mice, although the age of those mice was not specified (40).

In Wistar rats, males exhibited a higher number of proliferating cells than females and this sexual dimorphism was already established before puberty (41). The higher proliferation at the level of the ventricle does not lead to a sex difference in the density of newly generated cells in the MOB, but only in the volume of the granular layer in the anterior part of the AOB, larger in males than in females [(42); Table 1].

**Table 1:** Sexually dimorphic features in the subventricular zone-olfactory bulb (V-SVZ-OB) system.

Model	Feature	Higher in:	Where	Reference
Prepubertal	Proliferation rates	Males	V-SVZ	(41)
Wistar rats	Volume of the granule cell layer	Males	Anterior AOB	(42)
	Newly generated cells	Males	Anterior AOB	(42)
Peripubertal mice	Apoptotic cells	Males	V-SVZ	(33)
P60 C57/BL6 mice	Newly generated cells	Males	AOB	(34)
P90 C57/BL6 mice (estrous and pregnancy)	Proliferation rates SOX2 + progenitors	Females Females	V-SVZ, RMS, OB V-SVZ, RMS, OB	(33) (34)
P180-P240 C57/BL6 mice	Proliferation rates	Females	V-SVZ, RMS, OB	(39)
P180-P240 BALB/c mice	Proliferation rates	Males	V-SVZ, RMS, OB	(39)

Beside the cells belonging to the neurogenic linage, neural stem-cell niche encompass other structures, namely blood vessels (43, 44), microglia (45, 46), and choroid plexus (47). Both blood vessels (48), microglia (49, 50), and choroid plexus (51) are deeply affected by sex steroids. These structures, thus, may mediate the effect of sex steroids on adult neurogenesis (Figure 1).

Moreover, V-SVZ neurogenesis may also be modulated in a trans-synaptic way by other neuronal circuits which may be sensible to sexual steroids, e.g., serotonin or dopamine system (52, 53), and cholinergic neurons (54).

In general, estrogens are neuroprotective and stimulate differentiation and proliferation while progestins and androgens stimulate differentiation and cell survival (21). However, the V-SVZ-OB system has its unique features. In conclusion, a number of factors can affect adult neurogenesis in the V-SVZ-OB system, and it is likely that some of them are sexually dimorphic and change throughout lifetime. In this picture, the endocrine system may play a key role.

## Hormonal Regulation of V-SVZ Neurogenesis in Adult Females

Circulating hormone levels dramatically change during the life of female rodents, during both estrous cycle and pregnancy. These changes may affect neurogenesis. In particular, E_2_ levels control the estrous cycle, pregnancy, and sexual behavior (32, 55).

Since OB has a key role in mother’s offspring recognition, it is not surprising that the rate of neurogenesis in V-SVZ transiently increase during pregnancy (56). Indeed, two peaks of cell proliferations were observed at gestation day 7 and at postpartum day 7, while at delivery the neurogenic rate is similar to matched aged virgin females (56). The first peak is evident also in females mated with sterile males, so it depends on maternal hormonal levels rather than on the embryo. However, this effect is mediated by prolactin rather than E_2_ or progesterone (56, 57). However, E_2_ may have an indirect role, since it stimulates prolactin release (58).

In the adult female mouse, E_2_ has an inhibitory effect on V-SVZ-OB neurogenesis in both V-SVZ and OB. First, it decreases cell proliferation in the V-SVZ in different models. The number of proliferating cells in the V-SVZ is lower during estrus, than proestrus (39). Moreover, in ovariectomized females, acute E_2_ supplementation for one day, with a dose comparable to the estrus, decreases cell proliferation in the V-SVZ (59). On the other hand, this effect was not detected by long-term treatment [3 weeks (60)] or with a lower dose of E_2_ (61), comparable with diestrus (62). Differences in the effect of ovariectomy may be due to an interplay of many component of the neural stem-cell niche. In fact, ovariectomized mice express both ERα and ERβ, but E_2_ supplementation selectively upregulates ERβ (51). T metabolite 5α-dihydrotestosterone (5αDHT) decreases the expression of AR in the choroid plexus of ovariectomized mice (51).

Male pheromones stimulate the production of ovarian hormones (63) as well as the neurogenesis in adult females (35, 64, 65). However, E_2_ does not increase neurogenesis (66), nor cell proliferation in V-SVZ or neuroblasts density in OB, but it decreases cell survival in AOB, but not in MOB (24).

In the OB, E_2_ has different effects depending on the region. In the MOB, in adulthood rather than during development, E_2_ is able to impair the survival of newly generated cells (59) and MOB functionality (60). Interestingly, as demonstrated in aromatase-KO mice, developmental E_2_ has the opposite effect in the AOB: the absence of E_2_ during development decreases the survival of adult generated cells in the AOB. This phenotype can be reverted by adult E_2_ treatment. On the contrary, the lack of estrogens during development neither alters cell proliferation in the V-SVZ, nor its response to E_2_ (60).

**Figure 2:**
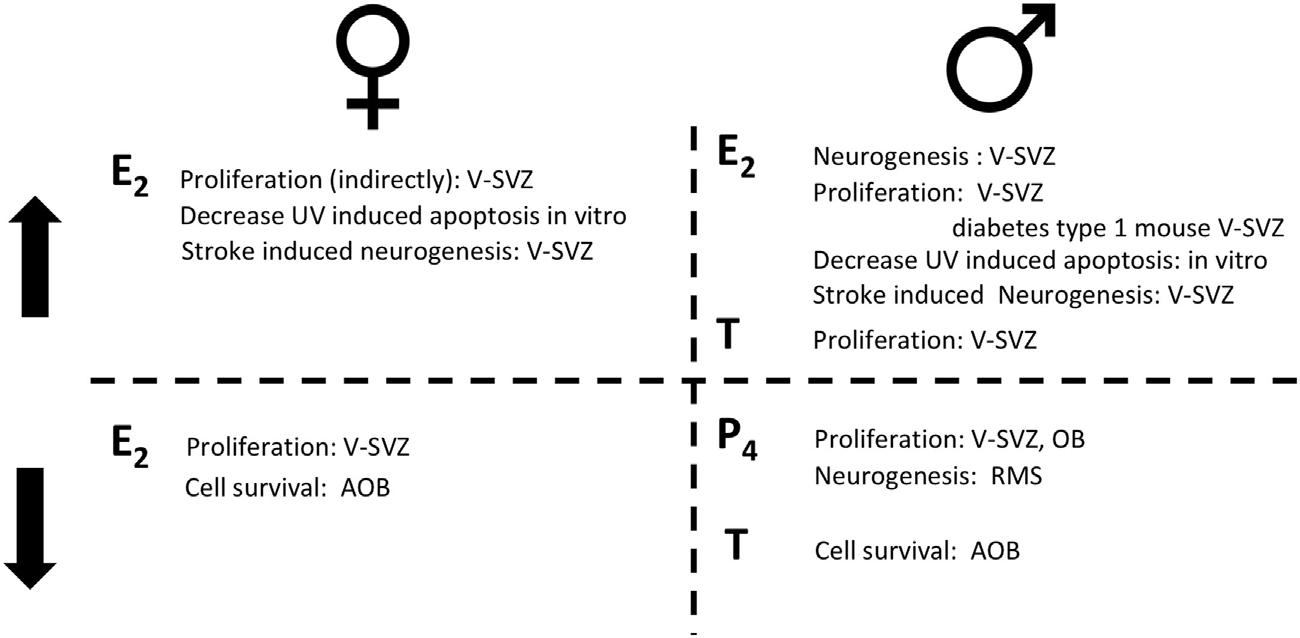
The role of sex hormones in adult neurogenesis for females (left) and for males (right). On the top, steroid hormones induce an increase (↑) in the reported actions; on the bottom steroid hormones induce a decrease (↓) in the reported actions. Estradiol (E_2_); progesterone (P_4_); testosterone (T).

In contrast to mice, the proliferation rate in the rat V-SVZ does not change during pregnancy, while it increases at delivery (67). As for mice, E_2_ role in female rat is highly debated. Proliferation in the V-SVZ is not affected neither by ovariectomy nor by acute T or E_2_ supplementation (41). No studies are available on the long-term effects of ovariectomy despite it deeply alter choroid plexus transcriptome which may indirectly affect the neural stem-cell niche (68). However, E_2_ decreases the survival of newly generated cells in the AOB, but not in the MOB [(29, 30); Figure 2; Table 2].

**Table 2:** Summary of the hormonal regulations in the subventricular zone-olfactory bulb (V-SVZ-OB) system of adult male and female rodents.

	Hormone	Effect	Where	Reference
**Hormonal regulation of V-SVZ neurogenesis in adult females**				
Mouse	Estrogen	↓ Proliferation	V-SVZ	(39)
		↑ Proliferation (indirectly)	V-SVZ	(56, 57)
		↓ UV-induced apoptosis	*In vitro*	(34)
		↑ Stroke-induced neurogenesis	V-SVZ	(61, 69, 70)
		↓ Cell survival	MOB	(59)
		↓ Functionality	MOB	(60)

Mouse, rat	5α-dihydrotestosterone	↓ Expression AR	Choroid plexus	(51)
		↓ Cell survival	AOB	(29, 30, 66)

**Hormonal regulation of V-SVZ neurogenesis in adult males**				
Mouse	Castration	↑ Proliferation	V-SVZ	(39)
		↓ Apoptosis	V-SVZ	(39)
	Estrogen	↓ UV-induced apoptosis	*In vitro*	(34)
		↑ Stroke-induced neurogenesis	V-SVZ	(61, 69, 70)

Type 1 diabetes mouse model		↑ Proliferation	V-SVZ	(71)
		↓ Cell survival	AOB	(72)

Rat	5α-dihydrotestosterone	↓ Expression AR	Choroid plexus	(51)
	castration	↑ Expression ERβ	Choroid plexus	(51)
		↓ Proliferation	V-SVZ	(41)
	Estrogen	↑ Proliferation	V-SVZ	(41)
		↑ Stroke-induced neurogenesis	V-SVZ	(73, 74)
		↑ DCX + cells after stroke	V-SVZ	(73)
		↓ Cell death	V-SVZ	(74)
	Testosterone	↑ Proliferation	V-SVZ	(41)
	Progesterone	↓ Proliferation	V-SVZ/OB	(75)
		↓ Neurogenesis	RMS	(75)

The different effects of E_2_ in mice and rats may be related with the lack of ERα and ERβ in the mouse V-SVZ (76) and with the presence of ERα receptor in the rat (28), although other pathways may be involved (21). For example, no information is available at the moment, concerning the expression of membrane ER (GPER) in rodent V-SVZ.

## Hormonal Regulation of V-SVZ Neurogenesis in Adult Males

The effect of sexual steroids is complex also in males. In fact, castration increased the number of proliferating cells and decreased the number of apoptotic ones in the V-SVZ of C57BL6 and SJL/J adult males, i.e., 6–8 months old (39).

Neurogenesis is influenced by pheromones related to aggressive (35) and paternal behavior (77, 78). In fact, the response to pheromones is sex specific and affected by hormonal levels. Indeed, female pheromones stimulate neurogenesis in adult males (64), although the survival of newly generated cells in the AOB in males does not change after opposite sex pheromones exposure, as in females (65). Interestingly, male pheromones as well as female ones, enhance proliferation in the V-SVZ of males (35), although it did not change the ratio of SOX2 cells among the BrdU labeled ones (64). However, low T levels feminize neurogenic response, increasing newly generated cell survival in the AOB, following male pheromone exposure, without affecting cell proliferation in RMS and V-SVZ, leading to attraction to male cues (72).

E_2_ have a neuroprotective effect on V-SVZ precursors. In fact, it is able to restore proliferation in a type 1 diabetes mouse model (71). Only a few choroid plexus genes are altered by castration in rats (68): ERβ expression increased when compared with sham operated rats (51), while ARs expression decreased after 5αDHT treatment (51).

Unlike in females, T or E_2_ are required for maintaining physiological neurogenic rate in the V-SVZ of peripubertal rats (41). In fact, the number of proliferating cells and the number of type C progenitors is restored by hormonal treatment in castrated rats, but this effect is restricted to the lateral wall of the V-SVZ (41).

Progesterone and its metabolites, decrease cell proliferation in the V-SVZ-OB of adult rats [2 months old (75)]. The number of newly generated cells in the final part of the RMS is decreased by progesterone metabolites. It is not clear, however, whether this effect is due to a reduction in cell proliferation, of cell survival or, less likely, in the migration rate (Figure 2; Table 2).

## Hormonal Regulation of V-SVZ Neurogenesis in Pathological Conditions

Beside an effect in physiological conditions, sex steroids may have a neuroprotective role after different insults. In fact, while no effect of sex steroid treatment was observed on cell death *in vitro*, E_2_ prevented apoptosis after UV insults in both male- and female-derived V-SVZ cells, whereas no T effect was reported [(34); Table 2].

Stroke induced an increase in the number of newly generated cells, which was significantly higher in females. As for UV-induced apoptosis, E_2_ enhances neurogenesis after ischemic stroke, *in vivo*, in mice of both sexes (61, 69, 79) and rats (73). This increase is present 96 h but not 24 h after stroke (61). The presence of ERα and ERβ, as well as AR is required for the stroke-induced neurogenesis in female mice, since it is abolished in transgenic mice lacking those receptors (69). Interestingly, those receptors are not directly expressed in the V-SVZ (61) suggesting that they may act indirectly through other cells.

Gonadal hormones are supposed to have a key role also in many diseases which display a different incidence and severity in the two sexes (80). V-SVZ neurogenesis may have a prominent role in some of them as: Parkinson disease (17, 81), Multiple sclerosis (82, 83), Alzheimer disease (84), autism (85), schizophrenia (86), and in psychiatric and cognitive disorders (87). However, only limited data are available on the effect of neuroactive steroids on the V-SVZ neurogenesis in those diseases. Moreover, many studies report controversial data on changes in the V-SVZ neurogenesis that may be related on the experimental model as in Parkinson disease (17).

## Concluding Remarks

Despite the huge amount of studies on adult neurogenesis in the V-SVZ-OB system, still few data focus on its regulation by steroids. The role of steroids on V-SVZ-OB neurogenesis is highly complex. Generally, neurogenesis is more affected by T in males, while E_2_ has a higher influence on females. However, the same hormone may determine a different effect depending on sex, age, strain, brain region, and neurogenic process. It is also possible that the different extent of V-SVZ-OB neurogenesis may reflects behavioral differences described among many strains of mice (88) as observed in other brain regions (89, 90). Those differences may be genetic (91, 92) or depend on a lack of maternal care during development (93). Profound differences exist between males and females. Some of them are actively determined by steroids levels in adults, while others are established during development. Moreover, sexual hormone’s levels changes along life. Important species-specific differences exist between different rodent models. Despite some similarities, adult neurogenesis is regulated by different factors in the V-SVZ-OB system compared with the SGZ of the hippocampus. Furthermore, different cell populations, or different steps of the neurogenic lineage may be sensible to a specific hormone.

The extent of adult neurogenesis in the V-SVZ-OB changes along with each of the above mentioned parameters. However, it is not clear which features are directly or indirectly involved. It is, thus, important to consider all those parameters altogether.

## Author Contributions

All the authors equally contributed to the search for the sources and to the writing of the manuscript.

## Conflict of Interest Statement

The authors declare that the research was conducted in the absence of any commercial or financial relationships that could be construed as a potential conflict of interest.
